# Mechanical Properties and Microscopic Mechanism of a Multi-Cementitious System Comprising Cement, Fly Ash, and Steel Slag Powder

**DOI:** 10.3390/ma16227195

**Published:** 2023-11-16

**Authors:** Yuzhi Zhang, Shujing Zhang, Qingke Nie, Liang Shen, Wei Wang

**Affiliations:** 1 Key Laboratory of Structural Health Monitoring and Control, Shijiazhuang Tiedao University, 17 North 2nd Ring East Road, Shijiazhuang 050043, China; 2 School of Safety Engineering and Emergency Management, Shijiazhuang Tiedao University, 17 North 2nd Ring East Road, Shijiazhuang 050043, China; 3 School of Civil Engineering, Shijiazhuang Tiedao University, 17 North 2nd Ring East Road, Shijiazhuang 050043, China; 1202101112@student.stdu.edu.cn (S.Z.); 1202101247@student.stdu.edu.cn (L.S.); 4 China Hebei Construction & Geotechnical Investigation Group Ltd., 555 Huai’an West Road, Shijiazhuang 050227, China; wangweizgyt@163.com; 5 Research Center for Geotechnical Engineering Technology of Hebei Province, 555 Huai’an West Road, Shijiazhuang 050227, China

**Keywords:** steel slag powder, cement based, hydration activity, mechanical properties, microstructure

## Abstract

The objective of this study was to reduce the stockpile of steel slag, which is a solid waste generated in the steelmaking process, and promote the resource utilization of steel slag powder (SSP) in construction projects. Experimental research was conducted on SSP and fly ash (FA) as supplementary cementitious materials. Composite cement paste samples were prepared to investigate the effects of the water-to-binder ratio and cement-substitution rate on the macroscopic mechanical properties, including the setting time, fluidity, flexural strength, and compressive strength of the prepared paste. The mineral composition in the raw materials was measured using X-ray diffraction (XRD), and a micro-morphological and structural analysis of the hydrated cementitious material samples was performed using scanning electron microscopy (SEM); the SEM and Image Pro Plus (IPP) image analysis techniques were combined for a quantitative analysis of the microstructure. The results showed that the addition of FA and SSP delayed the hydration of cement, thereby improving the flowability of the composite paste. Under the same curing age and cement substitution rate, the sample strength decreased with increasing water-to-binder ratio. Under the same water-to-binder ratio and curing age, the variations in the flexural and compressive strengths of the SSP group samples were inconsistent in the early and later stages, and the sample group with 20% SSP exhibited optimal mechanical strength in the later stage. The microscopic results showed that the needle-like AFt crystals in the hydrated pores decreased in number with the increase in the SSP content. The hydration products of the FA–SSP admixture, such as C–S–H gel and RO phase, acted as pore fillers in alkaline environments. When the water-to-binder ratio was 0.4 and the FA-to-SSP ratio was 1:1 to replace 40% cement, the performance of the hardened cement paste was the best among all the test groups containing both FA and SSP. This study provides a theoretical basis for the practical application of SSP and FA as cementitious materials in construction-related fields.

## 1. Introduction

Industrial solid waste is being produced at a rapid rate globally. To achieve carbon neutrality and reduce peak carbon emissions, improving the comprehensive utilization rate of such waste has become an important research direction. Steel slag is an industrial waste residue generated in the steelmaking process, and its emissions account for approximately 15% to 20% of crude steel production [1,2]. China’s steel production has consistently ranked first in the world for many years. The current cumulative stockpile of steel slag tailings is approaching 2 billion tons, and the amount of waste residue continues to increase annually, while the overall utilization rate remains only 20% to 30%, which is far below the planning requirements and limit level of developed countries [3,4].

In structural engineering, the application of steel slag aggregates is associated with a series of quality issues due to their poor stability [5,6]. In comparison, in the field of construction materials, a comprehensive utilization of steel slag powder (SSP) is more widely adopted [7,8]. Currently, the feasibility of using SSP as a construction material has been extensively studied [9,10]. Steel slag contains active mineral components, such as C_2_S and C_3_S, which impart the slag with certain cementitious properties, making it potentially useful in the production of concrete [11,12]. Wang et al. [13,14] found that SSP has weak early-stage cementitious properties and has little influence on the formation of hydration products in cement at the early stages; nevertheless, its promoting effect is mainly manifested in later-stage hydration. Liu et al. [15] found that silica fume can enhance the connection between the steel slag micropowders and the surrounding calcium silicate hydrate (C–S–H) gel, and the overall activity of the composite mineral admixtures can be improved with the increase in the silica fume content. Kourounis et al. and Shi et al. [16,17] found that blended cement with SSP exhibits a good performance but a slightly low strength. Zhao [18] reported that the strength and degree of hydration of blended cement paste can be significantly improved with the reduction in the particle size of the SSP.

The high hardness, fine particle size, and other characteristics of FA and its low price make it a suitable cement admixture [19]. Ytterdal [20] found that the use of FA and ground granulated blast furnace slag (GGBFS) increased the binding capacity and reduced the amount of free chloride available for corrosion initiation. Lee and Zeng [21,22] found that the use of FA as an alternative to cement resulted in low early strength but helped enhance the long-term strength and durability of concrete. Fang [23] found that the use of SSP and FA instead of cement as the raw materials for concrete could improve the workability of concrete and improve its later strength. In addition, the use of FA as a cement admixture for concrete could improve its mechanical strength and erosion resistance while reducing the discharge of solid waste, thereby reducing the negative impact on the environment [24]. Therefore, FA is considered an ideal alternative. However, few scholars have explored the combined effect of SSP and FA as the cementitious material substitute for cement on the mechanical properties and micro-mechanism changes of the paste.

With this background, this study conducted experimental research on the preparation of cementitious materials by jointly replacing cement with SSP and FA. Different water-to-binder ratios, replacement ratios, and total replacement rates were used as independent variables to design the proportions of the various components in the cement-based materials. The material properties were characterized using X-ray diffraction (XRD) and scanning electron microscopy (SEM). Furthermore, the effects of FA and SSP on the fluidity, flexural and compressive strengths, and microstructure of the cement paste were investigated; the synergistic effect of their combined use was explored, and the optimal replacement ratio and dosage of both the materials were determined. Our study can serve as a basis for improving the disposal level of steel slag waste while meeting the basic performance requirements of cementitious materials.

## 2. Experimental Scheme

### 2.1. Materials

The SSP used in the experiment was obtained from The New Materials Technology Co., Ltd. (located in Handan, China) in the form of a black powder, and its performance indicators met the requirements of the Chinese National Standard GB/T 20491-2017 [25]. The cement used in the preparation of the cement paste samples was P.O 42.5 grade cement, which meets the GB 175-2007 [26]. The FA was obtained from Boheng Mineral Products Trading Co., Ltd. (located in Shijiazhuang, China); the Grade II FA was selected, and the performance indicators met the GB/T 1596-2017 [27]. Ordinary tap water was used as the water supply. Table 1 presents the physical properties of the raw materials used. Table 2 presents the chemical composition of the FA and SSP.

### 2.2. Preparation of Cement Paste Sample

In this study, experimental research was conducted on the combined use of FA and SSP as the cementitious materials for cement. Table 3 presents the proportions and mixing ratios of the components in the cement paste samples. Under the condition that no water-reducing agent was introduced, two water-to-binder ratios (*w*/*b*) were selected: 0.40 and 0.45. Here, the subscripts x, y, and z in C_x_F_y_S_z_ indicate the amounts of cement, FA, and SSP, respectively. Apart from the control group experiment with pure cement (C_100_F_00_S_00_), to investigate the influence of FA and FA–SSP on the mechanical properties of the cement-based materials, the FA content in the single-FA admixture test was set to 20% (all percentages are by mass); this formed the second control group, denoted by C_80_F_20_S_00_. In the FA–SSP compound mixing test groups, the FA content was fixed at 20%, the SSP contents were set to 20%, 40%, 60%, 80%, and 100%, and the FA-to-SSP ratios were 1:1, 1:2, 1:3, 1:4, and 1:5, respectively, to increase the utilization rate of the SSP, respectively; the corresponding samples formed were denoted by C_60_F_20_S_20_, C_40_F_20_S_40_, C_20_F_20_S_60_, C_00_F_20_S_80_, and C_00_F_00_S_100_, respectively. When *w*/*b* was 0.45, the test group was denoted by C_100_F_00_S_00_^1^. To facilitate the study of the *w*/*b* effect on the properties of net cement paste samples, the cement, FA, and SSP were weighed and mixed proportionally. The mixing water was weighed and poured into the net cement paste mixer and mixed, and the net paste samples were placed in a standard environment (temperature: 20 ± 2 °C; relative humidity: >95%) for 24 h of curing and then demolded. The demolded net paste samples were kept in a standard maintenance box until the specified age, and the flexural and compressive strengths were tested at 3 d, 7 d, and 28 d.

### 2.3. Characterization and Testing

**Figure 1:**
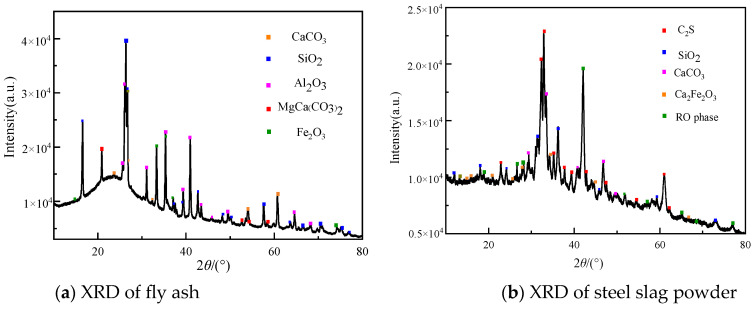
XRD patterns of the raw materials.

Mineral composition of raw materials (SSP and FA). The mineral composition of the SSP was determined using an X-ray diffractometer. The Smart Lab X-ray diffractometer (Model ZSX Prinmus IV, the manufacturer is Beijing Zhonghe Venture Technology Development Co., Ltd., which is located in Beijing, China.) has a scanning speed of 1 (°)/min, a step size of 0.02°, a scattering angle of 2θ, an angle range of 0–90°, and a power of 4 kW. The XRD pattern analysis and data processing were performed using a combination of MDI Jade 6.5 software analysis and Origin Pro2022 mapping, as shown in Figure 1. The FA mainly included common SiO_2_, Al_2_O_3_, and Fe_2_O_3_. The SSP mainly included C_2_S, CaCO_3_, and SiO_2_ and contained a certain amount of RO phase, which is a solid melt formed by magnesium, iron, and manganese oxides, that is, MgO, FeO, and MnO. As an inert mineral, the RO phase is a component that affects the performance of the SSP mixed into the cement base.

Determination of cement paste fluidity, setting time, and stability: In accordance with GB/T 1346-2011 [28], the setting time of the cement paste was determined using a Vicat apparatus, and a stability test was conducted using the Reynold’s method. In accordance with the GB/T 8077-2012 “Concrete admixture test method”, a fluidity test was conducted on the net cement paste [29], in which the paste flow was determined to characterize the different SSP admixtures in the cementitious test groups.

Mechanical property test on the net cement paste: In accordance with the GB/T 17671-2021 “Test method for cementitious sand strength (ISO method) [30]”, the flexural and compressive strengths of the cement–FA–SSP ternary composite paste were tested using a cementitious sand flexural and compressive testing machine (TYE–300D type) after curing periods of 3 d, 7 d, and 28 d. The sample dimensions were 40 mm × 40 mm × 160 mm, and the loading rate was 2.4 kN/s. The test error in the strength measurement of an average of six samples in each group should be less than 15%. If the error was not within this limit, the samples needed to be remade and retested.

Cement-based material microstructure testing with SSP addition: After the compressive strength test, the broken samples aged for different durations (3, 7, and 28 days) were collected and then soaked in anhydrous ethanol to terminate the hydration reaction. A scanning electron microscope (Phenom Pure type) was used to observe the micromorphology of the hardened paste surface in the high-vacuum mode. The Phenom Pure-type SEM equipment is equipped with a CeB6 filament, which has a lifespan of 1500 h. It operates at an accelerating voltage of 5 keV and has a maximum magnification of 30,000× with a resolution greater than 30 nm. The vertical distance from the irradiation position to the sample surface varies with the different magnifications, and the working distance is in the range of 2–45 mm.

To study the microstructural similarities and differences in the cementitious systems with different FA–SSP mixtures, the image processing software Image-Pro Plus 6.0 (IPP) was used to perform black-and-white binary processing on the SEM images. The characteristic parameters, such as the pore area, number of pores, pore diameter, and roundness, were extracted from the images. Finally, the microstructures of the hardened slurries of each experimental group were quantitatively analyzed.

## 3. Analysis of Mechanical Results

### 3.1. Fluidity

**Figure 2:**
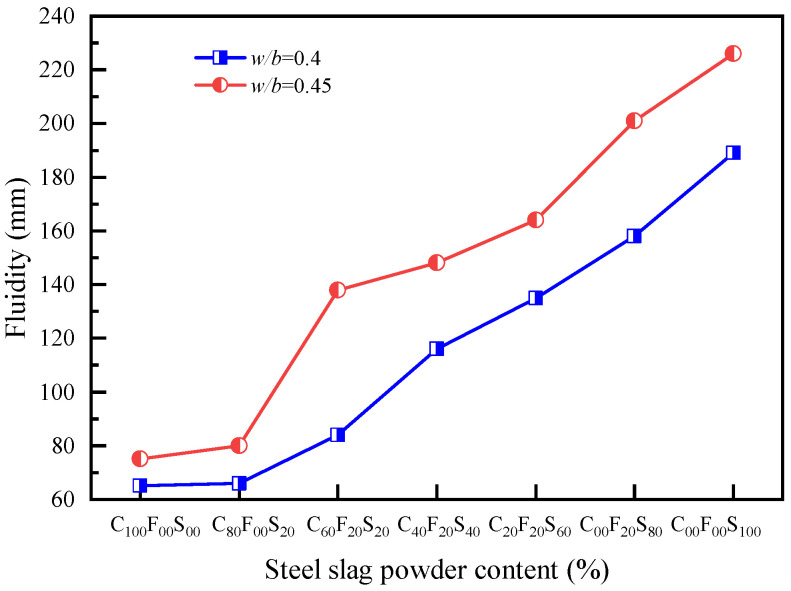
Fluidity of a ternary composite paste system containing cement, FA, and SSP.

**Figure 3:**
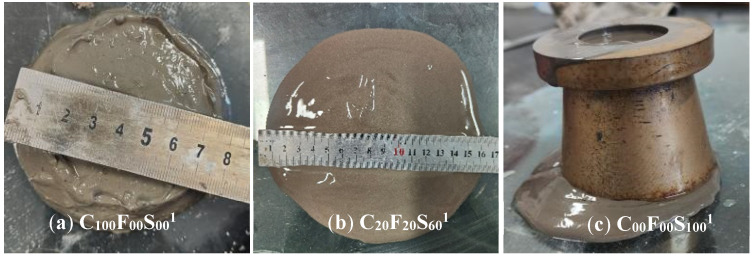
Influence of SSP on the fluidity of the composite paste.

Figure 2 shows the fluidity test results of the cement paste with different amounts of FA and SSP. C_100_F_00_S_00_ indicates that the FA and SSP amounts are both 0%, i.e., this group was the pure cement control group. As shown in Figure 2, with different water-to-binder ratios, the experimental groups with a *w*/*b* of 0.45 had a higher overall fluidity than the experimental group with a *w*/*b* of 0.40. However, the FA and SSP admixture had a similar impact on the changes in the fluidity of the cement paste. With the increase in SSP, because of the inert substance and low activity, the fluidity increased linearly. The combined effect of FA and SSP resulted in a paste fluidity that was similar to that of pure cement, thus playing a role in regulating the fluidity of the cementitious materials. Consistent with the experimental results obtained by Liu et al. [31], the addition of ordinary SSP increased the workability of the mortar. Under the same *w*/*b*, taking a *w*/*b* of 0.45 as an example, the fluidity of the cement paste with 20% of FA alone increased by 5 mm compared with that of C_20_F_20_S_60_^1^ (75 mm). The fluidity of C_60_F_20_S_20_^1^ was 138 mm, which increased by 63 mm compared with that of C_100_F_00_S_00_^1^, and 58 mm compared with that of C_80_F_20_S_00_^1^. However, the fluidity of C_20_F_20_S_60_^1^ was 164 mm, which was 89 mm greater and 2.19 times that of C_100_F_00_S_00_^1^. Therefore, both the FA alone and FA–SSP mixture could increase the fluidity; however, compared with the SSP, the mixing of FA further enhanced the effect of FA on the regulation of the fluidity of the cement paste. In terms of the test results, as shown in Figure 3c, for C_100_F_00_S_100_^1^, that is, when the SSP content was 100%, there was water secretion in the paste, mainly due to the lower water absorption of the SSP relative to the cement. In comparison, the steel slag had a lower activity, and it was only involved in the reaction when the aging duration was longer [32]. Thus, the combination of FA and SSP promoted the fluidity, which relatively increased the water content in the paste, and after reaching a certain admixture content, the paste flow was too high. At this time, the effect of SSP on improving the fluidity of the cement paste was unfavorable.

### 3.2. Setting Time and Stability

It is crucial to ensure that a cementitious material remains in the plastic state for a sufficient period during construction. Therefore, the setting time of the composite paste should not be excessively long or short. According to the GB 175-2007 standard, the initial setting time of silicate cement should not be less than 45 min, and the final setting time should not be greater than 600 min [25]. Under the two water-to-binder ratios, the ratio of FA and SSP was 1:3, that is, when the SSP content was 60%, the final setting time of the composite paste exceeded the setting time requirement by 865 min and 1070 min, respectively, which were 3.15 and 3.27 times the final setting times of C_100_F_00_S_00_ and C_100_F_00_S_00_^1^, respectively. The cement substitution amount was too high to lead to a long setting time, which was far from meeting the application requirements of practical engineering. For C_00_F_00_S_100_, there was an excessive amount of mixing water in the paste. After increasing the molding age, the inside of the paste was still not consolidated, and there was even a clear layer of water on the surface. Therefore, although the synergistic effect of FA and SSP can regulate the coagulation performance of cementitious materials to a certain extent, the SSP is not yet an equivalent substitute for cement as a cementitious material in terms of its performance. When the SSP content was 80%, the paste could not consolidate during the one-day aging. Although the water secretion of this sample was better than that of C_00_F_00_S_100_, it is far from meeting the application requirements of cementitious materials. Therefore, for an SSP dosage of 80% and above, the data were not quantitatively analyzed in this test.

**Figure 4:**
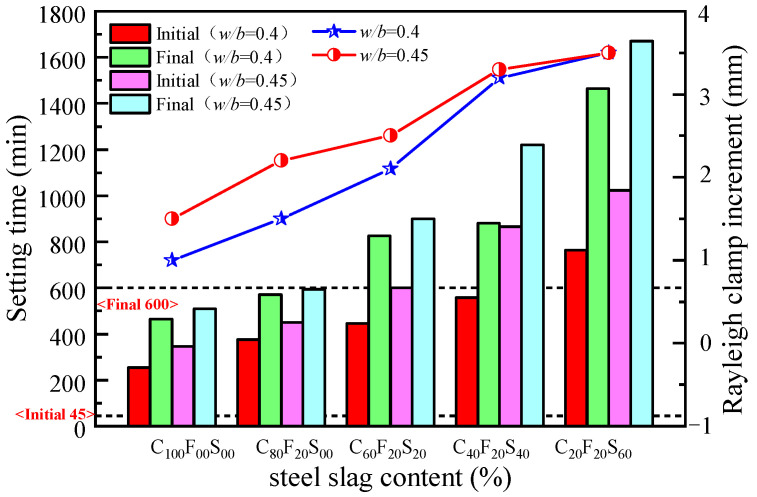
Setting time and stability of a ternary composite paste containing cement, FA, and SSP.

Different proportions of the composite paste with varying dosages were tested for their setting time and stability, as shown in Figure 4. Our experimental results showed that with the increase in *w*/*b*, the use of FA and SSP as admixtures prolonged the setting time of the sample. The setting time of the composite paste with the same *w*/*b* was faster than that of the net cement paste sample without FA and SSP. The setting time was prolonged when the amount of FA was fixed and the amount of SSP was increased. This was mainly because the cement was more active, the hydration reaction rate was higher, and the setting time was shorter when the amount of SSP was increased. Because the SSP has a significantly lower activity than ordinary silicate cement, it takes a longer time to form a stable structure inside the paste, and macroscopically, this is manifested in a prolonged coagulation time, and the SSP plays an inhibitory role. When the *w*/*b* was 0.4, the initial and final setting times of the C_60_F_20_S_20_ composite paste were 375 min and 570 min, which were 120 min and 105 min longer than those of the C_100_F_00_S_00_ cement paste, respectively. When the incorporation amount of FA and SSP was 1:2 for the substitute cement, the final setting time of the paste exceeded the requirements of the standard GB 175-2007 for general coagulation materials [26].

When *w*/*b* was 0.45, the time it takes for C_60_F_20_S_20_^1^ to begin setting and the duration until it reaches its final setting state were delayed by 105 and 85 min, respectively, compared with C_100_F_00_S_00_^1^. The combined content of FA and SSP was higher than 40%, and the initial and final setting times of the composite paste exceeded the setting time requirements of general cementitious materials. The reasons are analyzed as follows: SSP has an inhibitory effect on the coagulation of the composite paste, mainly because the components of SSP, namely, SiO_2_, CaCO_3_, and other chemicals, do not participate in the hydration reaction, and steel slag contains MgO, FeO, and other substances, the hydration activities of which are low, hindering the early hydration of cement. Therefore, the setting time of the composite paste was prolonged, consistent with the findings made by Zhao et al. [33].

**Figure 5:**
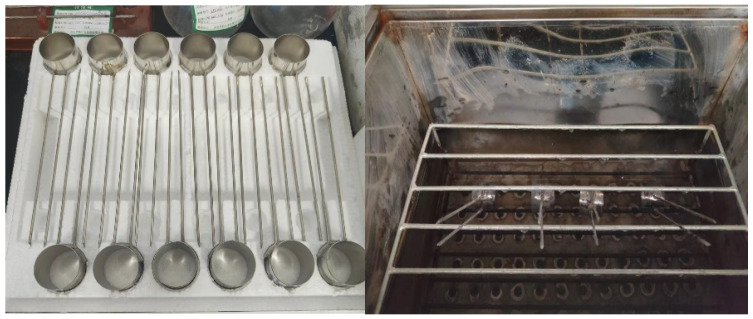
Rayleigh clamping boiling method for stability testing.

The stability of the different hardened slurries was determined using a Rayleigh’s clamp tester (LD–50) and a boiling chamber (FZ–31A, Hebei Huajian Metrology and Inspection Co., Ltd., Shijiazhuang, China), as shown in Figure 5. The results showed that the increment in Rayleigh’s clamp for each test group was within the safe volume stability range, i.e., within 5 mm. Within this range, compared with the control groups, an increase in the SSP admixture content led to a deterioration in the stability of the hardened paste. This may be due to the presence of free calcium oxide (f-CaO) in the SSP and the continuous hydration reaction in the hardened paste to form hydroxides, the increase in the solid-phase volume, volume expansion due to the internal stress generated in the paste, and increased paste volume in the presence of some insoluble substances in the SSP.

### 3.3. Flexural and Compressive Strengths

**Figure 6:**
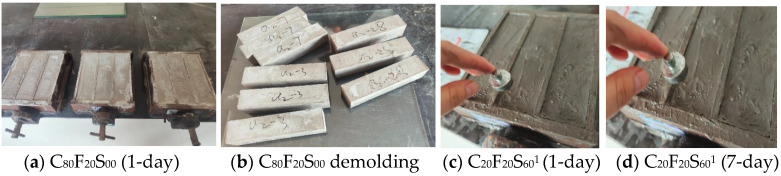
Comparison of test phenomena in the demolding state.

Table 4 presents the experimentally measured flexural and compressive strength data. When *w*/*b* was 0.45, C_20_F_20_S_60_^1^ was cured to a 1-day mold removal age. When the sample could not be sufficiently formed, it was extended to a two-day mold removal age, and its 7-day strength was almost 0 MPa. Figure 6 shows the test phenomenon, where the images a and b show the normal mold removal and molding state of the composite paste C_80_F_20_S_00_, and the figures c and d show the mold removal state of C_20_F_20_S_60_^1^. Clearly, the inside of the sample was still not completely consolidated, resulting in a low sample strength. Therefore, data for the test groups with 60%SSP and higher were not provided in this paper.

**Figure 7:**
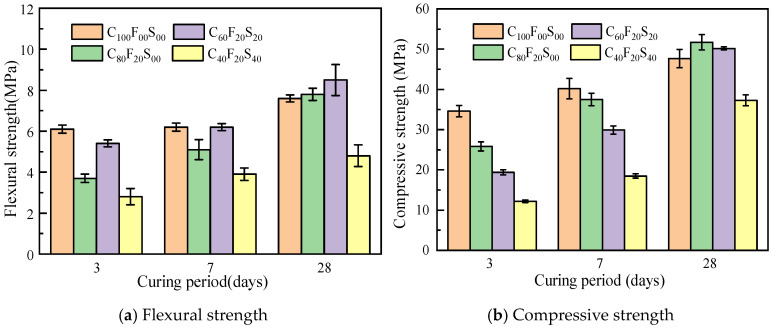
Mechanical strength of the composite paste after different curing periods (*w*/*b* = 0.4).

Figure 7 shows the test results of the flexural and compressive strengths of the composite paste at different curing periods with *w*/*b* of 0.4. When cured until the age of 3 days, the samples in the test group with only FA and FA–SSP blend exhibited lower flexural and compressive strengths than C_100_F_00_S_00_. By 7 days, the flexural and compressive strengths of the samples with only 20% FA (C_80_F_20_S_00_) increased to a certain extent, and the flexural strength had reached the same level as C_100_F_00_S_00_, whereas the compressive strength of the test groups with the FA–SSP blend was slightly lower. The compressive strength of C_100_F_00_S_00_ on day 28 was 47.7 MPa, whereas that of the test groups with only 20% FA exhibited a higher compressive strength, reaching more than 50 MPa, representing an increase of more than 8% compared with C_100_F_00_S_00_. The test group with an SSP–to–FA blend ratio of 1:1 and a total addition of 40% exhibited a compressive strength of 50.2 MPa on day 28, an increase of over 5% compared with the control group. Compared with C_100_F_00_S_00_, the mechanical properties of C_60_F_20_S_20_ on days 3 and 7 did not evidently improve; on day 28, a high compressive strength was reached, and the mechanical properties were better than those of the control group samples. This was mainly because the volcanic ash activity of FA needed some time to function, with the early hydration reaction primarily involving cement and water and FA and SSP participating in the hydration and accelerating the reaction in the middle and later stages. Additionally, the low reactivity of SSP hindered the early hydration of the blended paste. Consequently, within a certain dosage range, the test groups containing SSP showed a decrease in the early flexural and compressive strengths with increasing dosage, while the test groups with an optimal SSP content exhibited a higher strength in the later stage.

As shown in Figure 7, the overall length of the error bar is relatively uniform, which means that the test data are relatively stable, the discreteness is low, and the credibility is high. A variance test and significance analysis were performed on the flexural strength of the slurry, and the F and *p* values were 1.24 and 0.357 > 0.05, respectively, indicating a lack of significant difference between the test groups.

**Figure 8:**
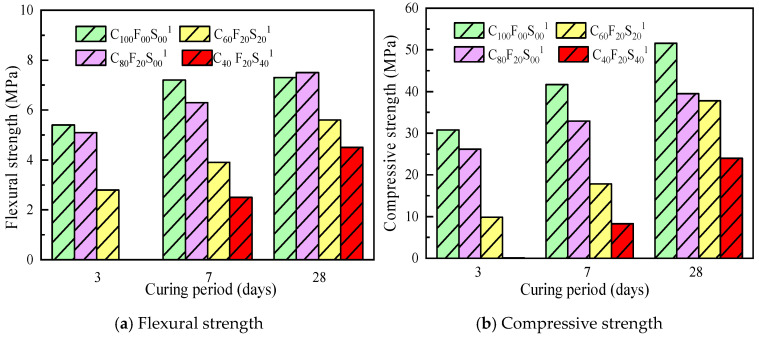
Mechanical strength of the composite paste after different curing periods (*w*/*b* = 0.45).

Figure 8 shows the variations in the mechanical properties of the paste cured for different ages with a *w*/*b* of 0.45. Clearly, the compressive strengths of both the FA and SSP samples were lower than that of C_100_F_00_S_00_^1^. The SSP amount was negatively correlated with the paste strength. Compared with C_100_F_00_S_00_^1^, which showed a compressive strength of 51.6 MPa on day 28, C_80_F_20_S_00_^1^ exhibited a strength of 39.5 MPa on day 28, a reduction of more than 2%, and the compressive strength of C_60_F_20_S_20_^1^ was 37.8 MPa on day 28, a reduction of approximately 3%; the compressive strength of C_40_F_20_S_40_ until day 3 showed a decrease of approximately 54% compared with C_100_F_00_S_00_^1^. When the dosage was too high, the amount of cement relative to that in the system decreased, resulting in an insufficient amount of hydration products. Therefore, the beneficial effect of FA and SSP on the mechanical properties of the cement paste was limited.

**Figure 9:**
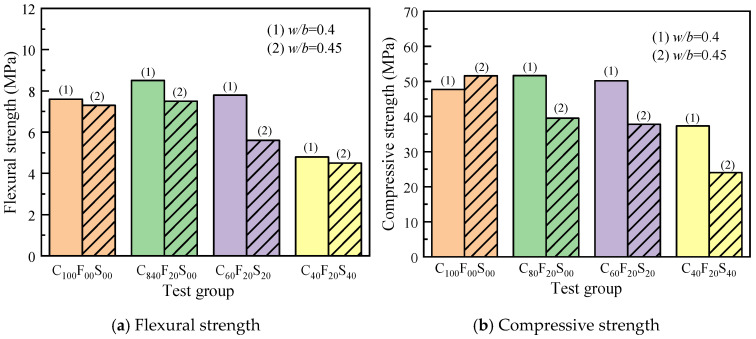
Influence of the water-to-binder ratio on the mechanical strength of the composite paste on 28 d.

Figure 9 shows the results of the effects of the *w*/*b* on the flexural and compressive strengths of the test groups at the same curing age. The 28 d compressive strength of the net cement paste samples with a *w*/*b* of 0.45 was higher than that of C_100_F_00_S_00_ with a *w*/*b* of 0.4, and the clean paste test groups containing SSP showed better mechanical properties than the test groups with a lower *w*/*b.*

**Figure 10:**
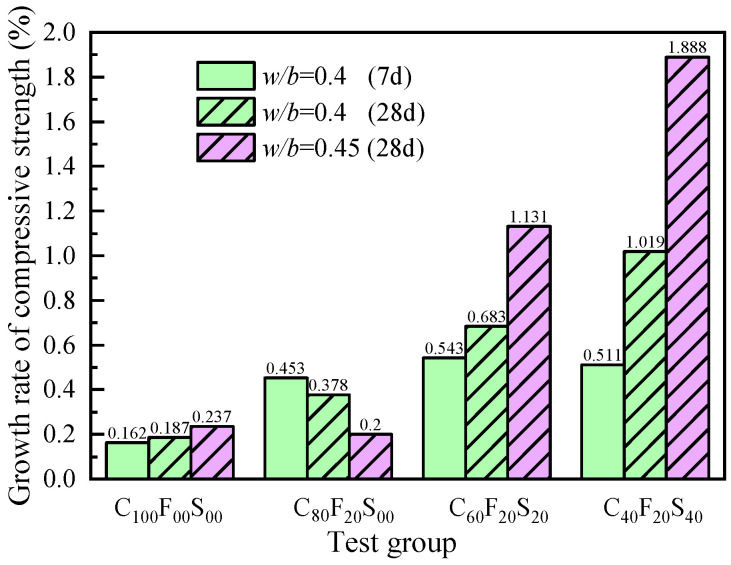
Growth rate of the compressive strengths of cement paste with different SSP contents.

Figure 10 shows the variation law of the growth rate of the compressive strength of the cement paste with the aging duration for cement slurries containing different SSP contents. Equation (1) can be used to calculate the growth rate of the compressive strength. At 7 and 28 days, the growth rates of the compressive strength of the pure cement paste were 16.2% and 18.7%. At a fixed 20% fly ash content and SSP contents of 0%, 20%, and 40%, the growth rates were 45.3%, 54.3%, and 51.1%, respectively, and the growth rates of the compressive strength of the 28 d paste were 37.8%, 68.3%, and 101.9%. When the *w*/*b* was 0.45, the growth rates of the 28 d compressive strength of C_60_F_20_S_20_ and C_40_F_20_S_40_ were 113.1% and 188.8%, respectively, and the later-stage strength increased significantly due to the delayed hydration caused by the addition of SSP, and within a certain admixture range, although the early-stage strength of the paste mixed with SSP increased by a small extent, the incorporation of SSP had a positive effect on the later-stage strength. This further indicated that the FA–SSP blend had a certain positive effect on the mechanical properties of the paste.

(1) $$\rho =(f_{2}-f_{1})/f_{1}$$

Here, ρ is the growth rate of the compressive strength in the current aging period; $f_{2}$ is the compressive strength at the current age, MPa; $f_{1}$ is the compressive strength at the previous age, MPa.

## 4. Microscopic Mechanisms

### 4.1. Micromorphology

**Figure 11:**
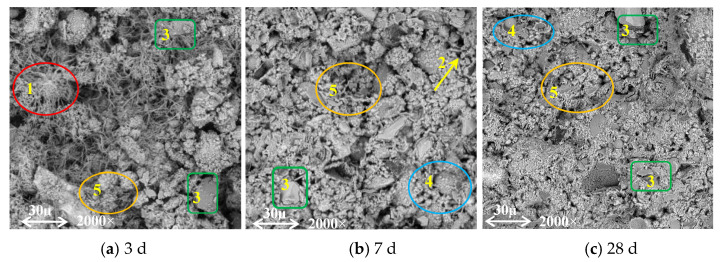
SEM images of the paste containing cement, FA, and SSP in the form of a multi-cementitious system. (1: C–S–H gel; 2: AFt crystals; 3: Ca(OH)_2_; 4: FA microspheres; 5: RO phase).

Figure 11 shows the micromorphology of C_40_F_20_S_40_ cured for different periods with a *w*/*b* of 0.4. Instead of using 60% cement, 40% SSP and 20% FA were added to participate in the hydration reaction. The chemical effect of this replacement is reflected in the fact that the hydration products of SSP and FA fill the pores in the hardened paste, and the unreacted admixture particles play the role of micro aggregate filling, that is, its physical filling role. Based on the SEM image, and the parameters and pore ratio extracted from it, the cured samples on day 28 were evidently denser than the cured samples on day 3. As shown in Figure 11a, the sample forms a flocculent C–S–H gel inside, a large number of short needle-like AFt crystals is deposited, and some unreacted raw material particles can be clearly observed. A comparison between Figure 11a–c shows that the AFt crystals significantly decrease in number with the increase in the curing time; however, some incompletely reacted particles remain. This is mainly because the inert mineral RO phase in the steel slag rarely participated in the hydration reaction, the amounts of gelling minerals C_3_S and C_2_S were low, the hydration induction period was longer, and the early hydration was very slow [34].

**Figure 12:**
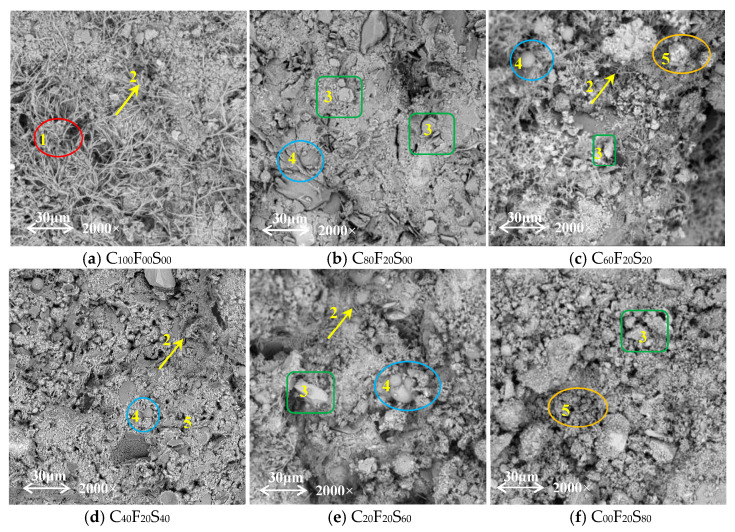
SEM images of hardened slurries cured for 28 days. (1: C–S–H gel; 2: AFt crystals; 3: Ca(OH)_2_; 4: FA microspheres; 5: RO phase).

Figure 12 shows the micromorphology of the hardened slurries with different SSP contents in each test group on day 28. Figure 12a shows that a significant amount of fibrous C–S–H gels and a large number of needle-like Aft crystals are generated inside the sample, compared with that shown in Figure 12a,b. In terms of the effect of a single FA admixture on the micromorphology of the cement paste, the micromorphology of the latter paste was more dense and continuous, and hexagonal plate-like Ca(OH)_2_ and spherical FA particles were clearly distributed on the surface; this is attributed to the high contents of active silicon and activated aluminum in the FA, which facilitated the generation of stable C–S–H and calcium aluminate hydrate (C–A–H). This resulted in a pronounced spherical cementitious effect, known as the microsphere effect, which improved the workability of the mixture and enhanced particle densification [34,35]. Figure 12c–f show the presence of a certain number of needle-like AFt crystals in the composite paste. These crystals filled the cracks and pores on the surface of the paste, and C–S–H on the surface and Ca(OH)_2_ embedded therein could also be observed, covering up the smaller spherical FA particles, and there were more plate-like C_3_S and circular-like C_2_S grains distributed around, and the hydration products generated also filled some of the microcracks.

### 4.2. Quantitative Analysis of the Microstructure

To reduce the difference in the scanning area of electron microscopy and the influence of magnification, the SEM images of the samples shown in Section 4.1 at resolutions of 500×, 1000×, 1500×, 2000×, 3500×, and 5000× were compared, and the overall change trend characteristics of the samples were obtained. The porosity and average roundness of the image particles and pores were selected as the basic research parameters. The porosity of the paste was calculated according to the extracted pore area and image area. The average roundness of the paste microstructure was calculated according to the extracted perimeter and area. The roundness here refers to the degree to which an object is close to being circular; the closer the roundness value is to 1, the closer the particle is to being circular; this can help analyze the morphological characteristics of the paste microstructure. The calculation formula is:

(2) $$R=\frac{p^{2}}{4\cdot \pi \cdot a}$$

Here, R is the roundness (mean), p is the perimeter of the sample, cm; and a is the pore area, cm^2^.

**Figure 13:**
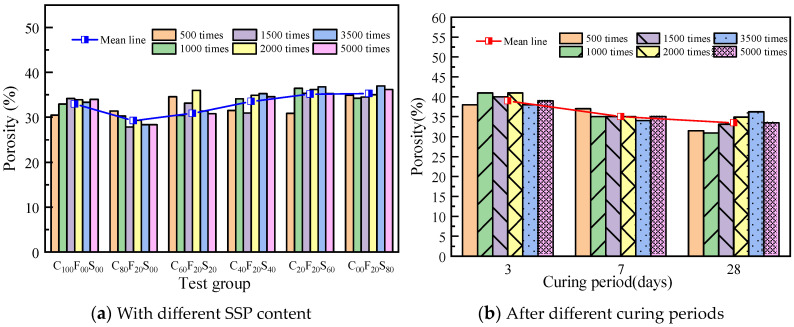
Change laws of the porosity of the composite paste.

Figure 13 shows the variation in the porosity of the paste samples; the overall porosity is in the range of 25–40%; from the trend in the average porosity line, it can be found that the SSP blend ratio has a relatively small effect on the porosity. Figure 13a shows the change in the porosity of the different test groups at different magnifications. The trend in the mean line shows that the porosity of the paste with FA alone is lower than that of the net paste samples, and the porosity of the samples increases with the increase in the SSP when the amount of SSP is fixed at 20% FA and the amount of SSP is more than 40%. In contrast, the aging period has a greater effect on the porosity. Figure 13b shows the change in the porosity of C_40_F_20_S_40_ after different curing periods under different magnifications. From the trend in the average porosity line change, it can be found that, as the curing age increases, the porosity of the sample decreases from 39.05% at 3 d age to 33.54% at 28 d age, with a change of 5.51%.

**Figure 14:**
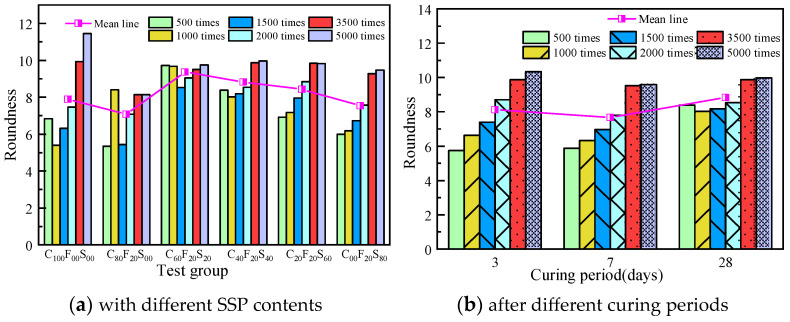
Variation in the average roundness of the hardened paste.

Figure 14 shows the variation in the mean roundness of the paste samples. Figure 14a shows the change in the average roundness of the samples with different FA–SSP compound dosages at different magnifications. The overall average roundness is in the range of 7–10, which is quite different from the standard value of 1, which indicates a circular shape, that is, the greater the difference between the obtained shape and the circular shape, the lower the impact of the replacement rate of the SSP on the roundness of the paste. The change law of the mean line shows that the roundness value of the paste with a single FA admixture was lower than that of C_100_F_00_S_00_, and the average roundness value of the paste was the highest in C_60_F_20_S_20_, which was 9.37. In the composite paste test groups, the greater the amount of SSP, the lower the roundness value. Figure 14b shows the change in the roundness of the paste sample cured after different periods at six magnifications. The histogram and change law of the mean line show that the overall average roundness of the paste is approximately 8.00, and the line chart shows that the change in the average roundness value is less, there is less volatility, and the effect of the curing age on the average roundness value of the samples has a nonlinear variation.

### 4.3. Analysis of Micromechanisms and Mechanical Properties

For the test groups in which the paste components contained both SSP and FA, the SEM microstructure was meso-analyzed using the IPP 6.0 software. Table 5 presents the porosity values. The porosity of C_40_F_20_S_40_ on day 3 was 0.390, while on day 7, its porosity was 0.347. Combined with the micromorphology and compressive strength results after different periods of each experimental group, it can be concluded that the porosity decreased with age, the number of needle-like AFt crystals decreased significantly in terms of the morphology, and there was a small number of incompletely reacted particles, mainly because the inert mineral RO phase in the SSP rarely participated in the hydration reaction. The contents of cementitious minerals C_3_S and C_2_S were low, the hydration induction period was longer, and the early hydration was slow. Therefore, the early strength of the paste was lower, and the number of needle-like AFt crystals decreased noticeably with increasing age. Based on the SEM image and the parameters and pores extracted from it, the micromorphology of the paste on day 28 was significantly more dense and continuous, thus improving the strength. This is also shown in Figure 13b, where the porosity decreases significantly with the increase in aging. The porosity of C_40_F_20_S_40_ on day 28 decreased by 5.51% compared with that of the sample on day 3, and the internal porosity of the paste was low, indicating that the internal density of the sample was high, and the compressive strength was higher. Combined with the mechanical test results, this conclusion is consistent with the variation law that the sample strength increased significantly with age.

At the same age, the macroscopic mechanism and microstructural change law of FA or SSP as the composite paste material instead of cement with different dosages were studied. Substituting an FA equivalent amount of 20% cement could increase the density and integrity of the microstructure of the sample. The porosity of C_80_F_20_S_00_ decreased by 0.02 compared with that of C_100_F_00_S_00_. At a fixed FA content of 20%, the internal porosity of the paste increased with the increase in SSP. When the SSP and FA content was set to 20%, the particles in the paste filled tightly, and the porosity of the cement composite paste was effectively reduced. Combined with the analysis of the previous test phenomenon, the flow performance and curing strength of the paste in C_60_F_20_S_20_ were effectively improved. When the fixed FA content was 20%, the SSP content was more than 40%; the porosity increased with the increase in the SSP content. The porosity of C_40_F_20_S_40_ was 0.335, which was 0.006 higher than that of C_100_F_00_S_00_ and 0.043 higher than that of C_80_F_20_S_00_. With the increase in the SSP content, the porosity increased, and the macroscopic strength showed a decreasing trend. Combined with the porosity of the microstructure obtained from the SEM, at this time, not all the particles in the SSP participated in the hydration reaction, and the excess water that did not participate in the hydration reaction formed blisters or waterways inside the paste. The hardening process formed pores, and the porosity increased, thereby decreasing the mechanical strength at the macroscopic level.

## 5. Conclusions

In summary, a multi-cementitious system comprising cement, FA, and SSP was experimentally studied. The following conclusions can be drawn from the results:

1. The remixing of SSP and FA could improve the fluidity of the cement paste, and the same amount of substitute cement under the synergistic effect of the two could extend the setting time of the cement paste. The amount of SSP exceeded 50%, resulting in an excessively long setting time of the sample and a lack of internal consolidation. This negatively impacts the mechanical properties and is not favorable for practical engineering applications. The Reynold’s test results showed that the stability of the cement paste slightly deteriorated when adding the SSP within a safe range.
2. The beneficial effects of FA and SSP on the mechanical properties of the net cement paste was limited, and the early flexural and compressive strengths of the test groups containing SSP showed a decreasing trend with increasing admixture content. Nevertheless, when *w*/*b* was 0.4, the late strength of C_60_F_20_S_20_ was higher than that of C_100_F_00_S_00_. When the SSP content exceeded 20% or *w*/*b* increased, the mechanical properties of the paste were adversely affected.
3. The SEM results showed that, compared with the control groups, the samples with the combined addition of SSP and FA exhibited a significant reduction in the number of needle-like AFt crystals with the increase in the aging period under the 28-day curing period. The samples cured for 28 d were significantly denser, which contributed to their internal density and compressive strength.
4. Substituting an equivalent FA amount to replace 20% cement could increase the density and integrity of the sample microstructure, and the internal porosity of the paste improved with an increase in SSP when the fixed FA admixture was 20%. The sample strength decreased with increasing SSP, and compared with the difference in the components, the effect of the curing age on porosity reduction became more evident.

Finally, the following aspects can be investigated in future studies:

1. Activation of SSP, mixing of SSP with other materials, and optimization of the mechanical properties of the FA–SSP–cement composite paste.
2. The cementitious material proposed in this study was applied as the cementitious material for steel slag concrete, investigating the influence of the FA–SSP–cement composite paste on the strength and durability of the steel slag concrete.
3. The long-term durability properties of composite pastes mixed with SSP and FA can be evaluated, e.g., erosion resistance, volumetric stability at longer ages (90, 180, and 365 days), permeation resistance, and frost resistance.

## Figures and Tables

**Table 1:** Physical properties of the raw materials used in this study.

Material	Density (g/m^3^)	Specific Area (m^2^/kg)	Water Content (%)	Loss on Ignition	Mobility Rate (%)	Activity Index (%)	Setting Time (min)	Stability (Reynold’s Method)
Cement	3.1	380	0.02	4.0	-	-	Initial = 194 Final = 315	Qualified
FA	2.1	350	0.9	≤6.5%	-	-		Qualified (≤4.5)
SSP	3.3	436	0.04	3.7	108	7 d = 70 28 d = 81	-	Qualified

**Table 2:** Chemical composition of the raw materials used in this study.

Material/wt.%	SiO_2_	Al_2_O_3_	Fe_2_O_3_	CaO	MgO	SO_3_	f-CaO	K_2_O	Cl^–^	MnO	Undissolved Substance	Total
FA	40	30	4.2	10	2.5	≤2.5	≤0.95	1.1	-	-	-	>97.8
SSP	19.48	6.25	21.18	32.7	6.01	0.25	2.41	-	0.02	1.90	7.3	>97.5

**Table 3:** Design of the mix proportions of cement paste.

Group	Water-to-Binder Ratio (*w*/*b*)	Mass Fraction/%			Total (SSP + FA)
		Cement	FA	SSP	
C_100_F_00_S_00_	0.40	100	0	0	0
C_80_F_20_S_00_		80	20	0	20
C_60_F_20_S_20_		60	20	20	40
C_40_F_20_S_40_		40	20	40	60
C_20_F_20_S_60_		20	20	60	80
C_00_F_20_S_80_		0	20	80	100
C_00_F_00_S_100_		0	00	100	100
C_100_F_00_S_00_^1^	0.45	100	0	0	0
C_80_F_20_S_00_^1^		80	20	0	20
C_60_F_20_S_20_^1^		60	20	20	40
C_40_F_20_S_40_^1^		40	20	40	60
C_20_F_20_S_60_^1^		20	20	60	80
C_00_F_20_S_80_^1^		0	20	80	100
C_00_F_00_S_100_^1^		0	00	100	100

**Table 4:** Flexural and compressive strengths of composite paste.

Water-to-Binder Ratio (*w*/*b*)		Flexural Strength (MPa)			Compressive Strength (MPa)		
		3 d	7 d	28 d	3 d	7 d	28 d
0.40	C_100_F_00_S_00_	6.1	6.2	7.6	34.6	40.2	47.7
	C_80_F_20_S_00_	5.4	6.2	8.5	25.8	37.5	51.7
	C_60_F_20_S_20_	3.7	5.1	7.8	19.4	29.9	50.2
	C_40_F_20_S_40_	2.8	3.9	4.8	12.2	18.5	37.3
	C_20_F_20_S_60_	-	-	3.5	-	5.4	8.3
0.45	C_100_F_00_S_00_^1^	5.4	7.2	7.3	30.8	41.7	51.6
	C_80_F_20_S_00_^1^	5.1	6.3	7.5	26.2	32.9	39.5
	C_60_F_20_S_20_^1^	2.8	3.9	5.6	9.8	17.8	37.8
	C_40_F_20_S_40_^1^	0	2.5	4.5	0.1	8.3	24
	C_20_F_20_S_60_^1^	-	-	-	-	-	-

**Table 5:** Comparative analysis of the mechanical performance index and microstructural parameters.

		C_100_F_00_S_00_	C_80_F_20_S_00_	C_60_F_20_S_20_	C_40_F_20_S_40_	C_20_F_20_S_60_
Compressive strength (MPa)	3 d	34.6	25.8	19.4	12.2	-
	7 d	40.2	37.5	29.9	18.5	5.4
	28 d	47.7	51.7	50.2	37.3	8.3
Porosity	28 d	0.359	0.292	0.309	0.335	0.351

## Data Availability

The data presented in this study are available on request from the corresponding author.

## Nomenclature

ρ	Growth rate of the compressive strength in the current aging period;
$f_{2}$	Compressive strength at the current age, MPa;
$f_{1}$	Compressive strength at the previous age, MPa;
R	Roundness (mean);
p	Perimeter of the sample, cm;
a	Pore area.
